# Real time extended range prediction of heat waves over India

**DOI:** 10.1038/s41598-019-45430-6

**Published:** 2019-06-21

**Authors:** Raju Mandal, Susmitha Joseph, A. K. Sahai, R. Phani, A. Dey, R. Chattopadhyay, D. R. Pattanaik

**Affiliations:** 1 Indian Institute of Tropical Meteorology, Pune, India; 2 Department of Atmospheric and Space Sciences, Savitribai Phule Pune University, Pune, India; 3 Centre for Prototype Climate Modelling, New York University Abu Dhabi, Abu Dhabi, UAE; 4 India Meteorological Department, New Delhi, India

**Keywords:** Atmospheric dynamics, Environmental impact

## Abstract

Heat waves over India occur during the months of March-June. This study aims at the real-time monitoring and prediction of heat waves using a multi-model dynamical ensemble prediction system developed at Indian Institute of Tropical Meteorology, India. For this, a criterion has been proposed based on the observed daily gridded maximum temperature (Tmax) datasets, which can be used for real-time prediction as well. A heat wave day is identified when either (1) Tmax (a)≥ its climatological 95^th^ percentile (calculated from daily values during March-June and for 1981–2010), (b) >36 °C, and (c) its departure from normal is >3.5 °C, Or, (2) when the Tmax >44 °C. Three heat wave prone regions, namely, northwest, southeast and northwest-southeast regions are recognized and heat wave spells of minimum consecutive six days are identified objectively for each region during 1981–2018. It is noticed that the prediction system has reasonable skill in predicting the heat waves over heat wave prone regions of India. Forecast verification of heat wave spells during 2003–2018 reveals that the prediction system has great potential in providing overall indication about the onset, duration and demise of the forthcoming heat wave spell with sufficient lead time albeit with some spatio-temporal error.

## Introduction

The Intergovernmental Panel on Climate Change (IPCC), in its 4th assessment report, states that *the type, frequency and intensity of extreme events are expected to change as the Earth’s climate changes*, and these changes could occur even with relatively small mean climate changes^1^. As extreme weather events are expected to occur more frequently and become more intense, the socioeconomic costs of those events are likely to increase as well^2^. Heat waves (HWs) are one of the hazardous extreme temperature events. Extremes of heat have broad and far-reaching impacts such as significant loss of life, health issues, and increased economic costs in transportation, agricultural production, energy and infrastructure.

In India, the HWs occur during the summer season months, March to June (MAMJ), mainly over north, northwest, central and the eastern coastal regions^3^. HWs over India are known to be linked with the climate mode such as El-Niño-Southern Oscillation or ENSO^4^. There is a noticeable increase in the frequency, persistency and spatial coverage of the HW days (based on IMD criteria, see Methods section) in the recent years and they are found to be more than average during the years succeeding El-Niño^3^. Studies^5^ indicate that HWs over India are associated with the anomalous sub-tropical persistent high with anti-cyclonic flow, depleted soil moisture over Indian land and with the SST anomalies over the tropical Indian and central Pacific Oceans. HWs can be classified into two types^6^: (1) those occur over the north-central India, which are associated with anomalous blocking over the North Atlantic Ocean and (2) those occur over the coastal eastern India, which are associated with the anomalous baroclinic Matsuno-Gill response to the anomalous cooling in the Pacific. Sometimes, HWs develop *in-situ*^7^. The analysis of daily maximum and minimum temperatures data of 121 stations over India during 1970–2005 has shown that the frequency of occurrence of hot days and hot nights have widespread increasing trend^8^. Recent studies suggested that, HWs in India will become more frequent and populations are especially vulnerable to these extreme temperatures^9,10^. The frequency of concurrent day and night-time HWs in India has increased in large part of western and southern parts during the post-1984 period^11^. HWs are expected to be more in the years succeeding the years with deficit summer monsoon rainfall, as in the case of year 2015^12^. Using a HW index based on daily maximum temperature (Tmax), researchers^13^ have shown that, the frequency of severe HWs will rise by 30 times under the increase in global mean temperature by the end of 21st century.

Although there are many studies on the mechanisms and future projections of HWs, very few studies^14–17^ have focused on the prediction of such events. There are no studies that focus on real-time prediction of HWs. Considering the impacts of such events across various sectors of the society, such as health, agriculture etc., there is a need to develop new strategy/criteria for the real time monitoring and prediction of such silent disastrous events over Indian region. The real-time Extended Range Prediction (ERP) (i.e. 2–3 weeks in advance) from skillful dynamical models can disseminate useful, understandable and timely information of such deadly events and can help reduce the adverse effects to a greater extent.

The present study proposes a criterion based on the daily gridded Tmax data for the real-time prediction of HWs over India on extended range during the summer season, MAMJ. A Multi-Model Ensemble (MME) prediction system is used in the present study for the evaluation and verification of the proposed criteria. Details of datasets used, the proposed HW criterion and the methodologies for identifying the HW spells over different HW-prone regions are discussed in the Methods section.

## Results

### Identification of HW prone regions

**Figure 1:**
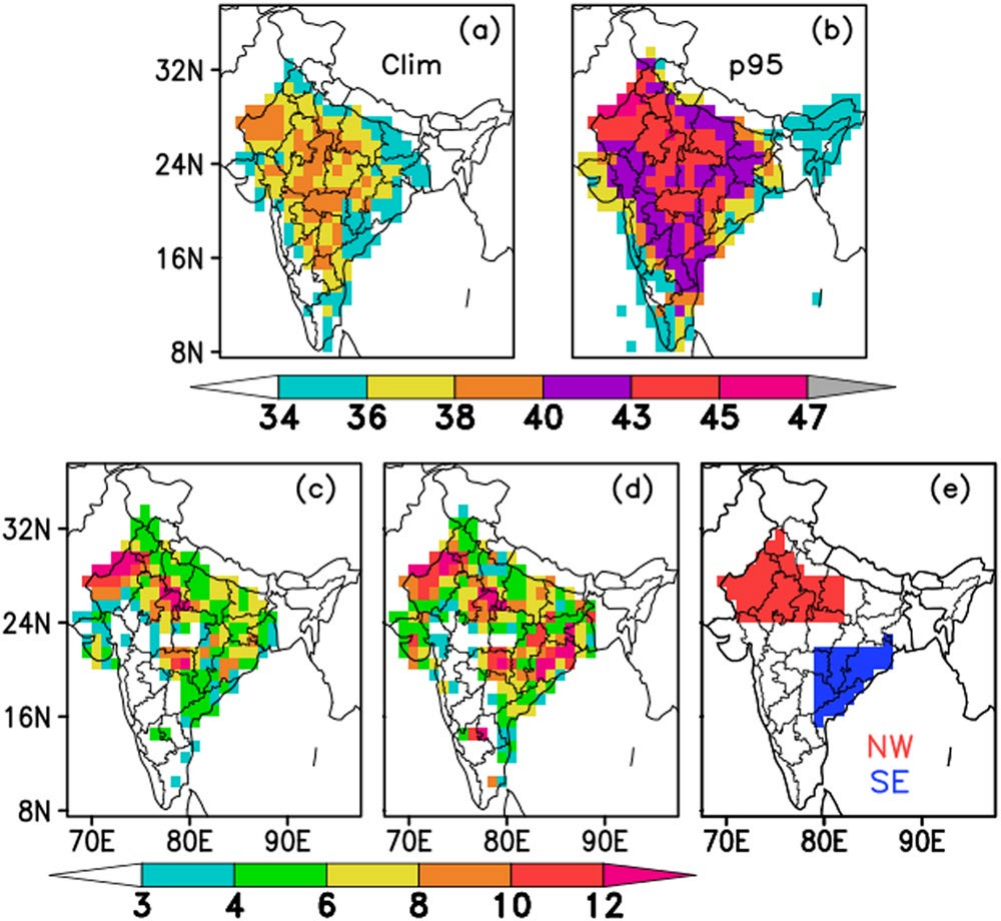
(**a**) Climatology and (**b**) climatological 95^th^ percentile of Tmax over the period 1981–2010 during MAMJ, (**c**) Average number of HW days during MAMJ for the period 1981–2017, (**d**) same as (**c**) but for the period 2001–2017, (**e**) NW (red shaded) and SE (blue shaded) HW prone regions.

Before defining the HW criteria, it is important to understand the observed spatial distribution of extreme temperatures. Figure 1a depicts the observed climatology of Tmax values during MAMJ for the period 1981–2010, whereas Fig. 1b represents the climatological extreme (95^th^ percentile values) of Tmax for the same period. It is observed that during MAMJ, Tmax values of more than 36 °C exist over northwest, central and parts of southeastern regions (Fig. 1a). Tmax values are less over the northeastern and southwestern parts of the country. Also, it is clear from Fig. 1b that over northwest, central, east and southeast coastal parts, the minimum 95^th^ percentile value of Tmax is 36 °C. Hence, based on these observed distribution of Tmax values, we have defined a HW criterion (please refer Methods section for more details about the proposed HW criterion) considering the actual (36 °C), departure from normal (3.5 °C) and 95^th^ percentile value of Tmax in gridded data. Based on the proposed HW-criteria, the average number of HW days during MAMJ over the period 1981–2017 and 2001–2017 have been calculated and placed in Fig. 1c,d respectively. It is noticed from Fig. 1c that, the north-west, central and south-eastern parts of India are the HW prone regions, having more than 4 HW days/year during MAMJ. It is interesting that in the recent years, the south east coastal region has become more vulnerable to HW events (Fig. 1d). It is also noted that, the West and east Rajasthan, Punjab, Haryana, Chandigarh, Delhi, West Madhya Pradesh, West and East Uttar Pradesh, Chhattisgarh, Orissa and Vidarbha, parts of Gangetic West Bengal, Coastal Andhra Pradesh and Telangana experience more than 6 HW days/year during MAMJ in the recent period (please refer Supplementary Fig. S1 for the regions). Many places in northwest and southeast coastal regions are having more than 8 HW days per season. Therefore, it is found that northwest, central and southeast coastal regions are mostly affected by the severe HW days in the summer season. On the other hand, the northeastern and southwestern parts of the country are less vulnerable to HWs.

Ratnam *et al*.^6^ identified two HW prone regions namely north-central India and coastal eastern India. However, the region chosen by them for the coastal eastern India was very small that covered only parts of coastal Andhra Pradesh and Telangana. It is found from Fig. 1d that the other southeastern coastal areas are also severely affected by HWs in the recent years. Therefore, considering the observed pattern in Figs. 1b–d, and to make the selection of the HW prone regions more authentic, the HW prone regions are identified as shown in Fig. 1e. The shaded regions shown in Fig. 1e are named as NorthWest (NW) and South-East (SE) region, marked with red and blue colours respectively.

### Skill of the ERP System in predicting extreme temperatures

**Figure 2:**
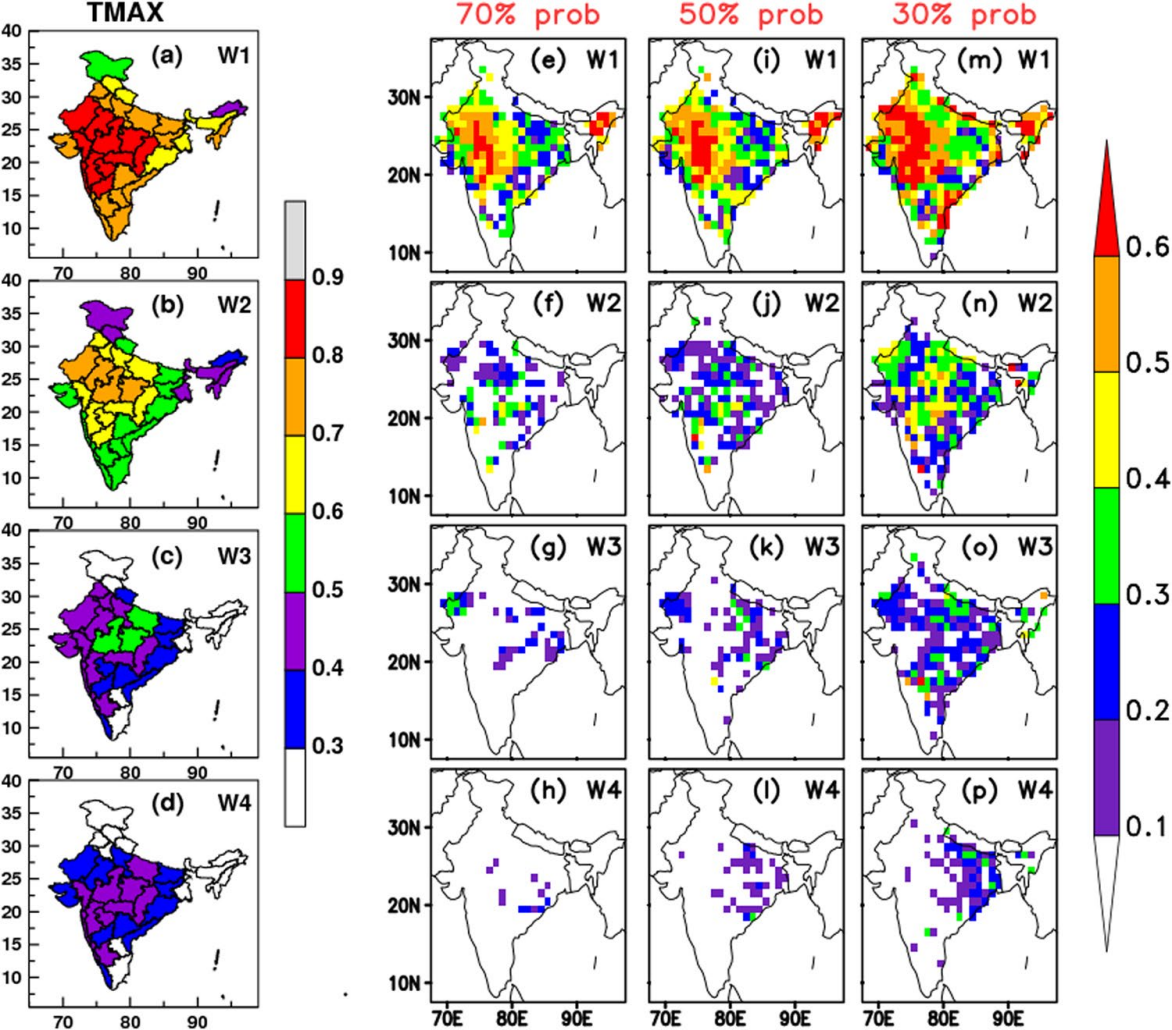
(**a**–**d**) ACC, (**e**–**h**) SEDI values for 70% HW probability, (**i**–**l**) SEDI values for 50% HW probability and (**m**–**p**) SEDI values for 30% HW probability for different week lead during MAMJ for the hindcast period 2003–2017. The left colour bar (for a-d panels) indicates the ACC values with 0.1 intervals, whereas the right colour bar (for e-p panels) represents the SEDI values with 0.1 intervals.

Many studies^5,6,13,15^ show the confidence to choose the Tmax parameter for the evaluation and prediction of the HW events over India. Hence, it is necessary to see the skill of the ERP system in predicting the Tmax during MAMJ before using it for HW prediction. Here, we use hindcast datasets for the period of 2003–2017 from CFSv2 based MME prediction system developed at Indian Institute of Tropical Meteorology (IITM), India and operationally run at India Meteorological Department (IMD), India (hereafter it will be termed as IITM-IMD ERP system) that generates real-time forecasts on a weekly basis (please refer Data subsection in Methods section for more details). Since the CFSv2 model is known to have bias in temperature^18^, the model forecasted Tmax values are bias-corrected before using them (please refer Methods section for details on the bias-correction methodology). Figure 2a–d shows the week-wise, i.e. for week-1 (wk1), week-2 (wk2), week-3 (wk3) and week-4 (wk4) leads (week refers 7days average), Anomaly Correlation Coefficient (ACC) (significant at 99.9% significance level) of Tmax during MAMJ for the hindcast period. Figure 2a,b indicates that the IITM-IMD ERP system has high values of ACCs for wk1 and wk2 leads over most parts of the HW prone regions identified (NW and SE). Over the central and northwest Indian regions, the significant ACC values are noticed even for wk3 and wk4 leads demonstrating the usefulness of the ERP system in HW prediction.

To further evaluate the skill of IITM-IMD forecast system in predicting the HWs events over Indian region, another index i.e. Symmetric Extremal Dependence Index (SEDI)^19^ has been used. SEDI is widely used for the deterministic forecast verifications of rare binary events. It can also be used for the verification of probabilistic forecasts for extreme events^20,21^, (if the probability is higher than a specified value, say 30%, 50%,90% etc. of the ensemble members predict the event). The main advantage of this index is, it is base-rate independent and is a function of H and F (see the Eq. (2) in Methods section); as lower base-rate has the effect of reducing confidence and stability^22^. Although the forecasts have to be calibrated before verification to produce a score suitable for comparing different forecasting systems^19^, no attempt has been made here to recalibrate the forecasts. While SEDI can be sensitive to model bias for rare events, the forecasts are used after removing the climatological bias (see the Methods section). To evaluate the SEDI values here we have considered our probabilistic HW forecasts as deterministic forecast by fixing some threshold probability values (e.g. 30%, 50% *and* 70%) for the occurrence of HWs. This means, it will consider the HW event as a binary event that whether the event is present with each of the above mentioned probabilities in ensemble forecasts. Figure 2e–p shows the week-wise (wk1 to wk4) SEDI values for 70% (Fig. 2e–h), 50% (Fig. 2i–l) and 30% (Fig. 2m–p) probabilities of HW occurrence. The positive SEDI values suggest that the forecast system is better-than-random, and higher positive values imply better skill. Figure 2(e,i,m) shows that the ERP system has very good skill in wk1 lead in predicting the HW events (even with 70% probability) over most parts of the HW prone regions. In wk2 lead (Fig. 2f,j,n) it shows reasonable skill up to 70% probability, with decreasing values for higher probabilities. With the 50% probability, even in wk3 lead it has a useful skill over few parts of NW and SE regions (Fig. 2k) for decision-making. With increasing lead and increasing probabilities, the skill becomes negligible (Fig. 2h). In wk4 lead, the ERP system has skill over few parts of eastern coastal region, but with 30% probability only (Fig. 2p).

**Figure 3:**
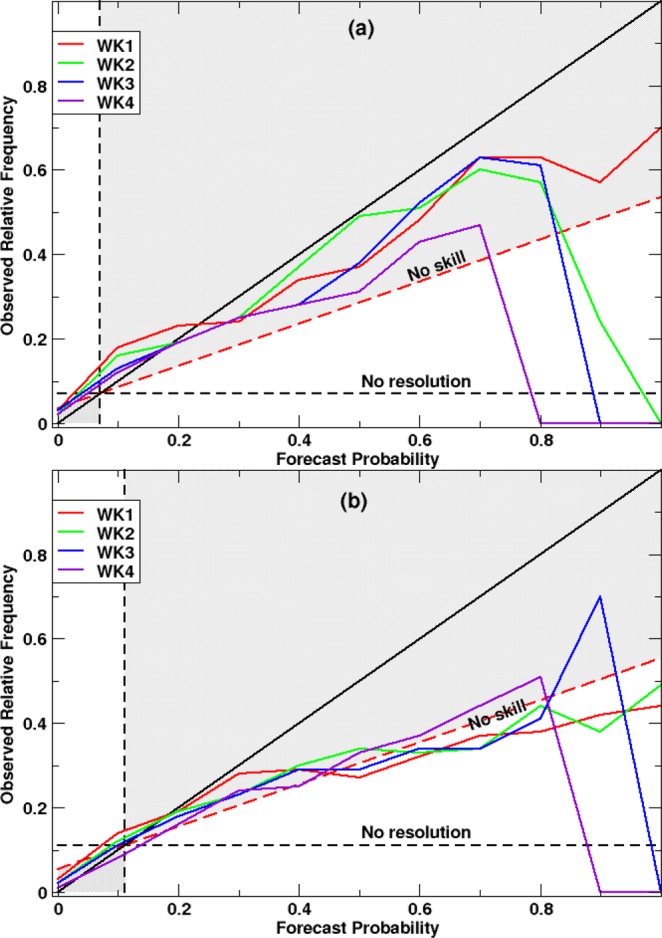
Reliability diagrams for the HW event (satisfying the proposed HW criterion) during MAMJ (2003–17) for two heat wave prone regions namely NW (**a**) and SE (**b**) for four different leads: week1 (red lines), week-2 (green lines), week-3 (blue lines) and week-4 (violet lines).

The reliability diagram (or the attributes diagram)^23^ measures how closely the forecast probabilities of an event (here, HW event) correspond to the actual chance of observing that event. The reliability diagram groups the forecasts into bins according to the issued probability along the horizontal axis. The frequencies with which the event was observed (i.e. the observed frequencies) to occur for these sub-group of forecasts are then plotted against the vertical axis. For perfect reliability, the forecast probability and the observed frequency should be equal and the point should lie on the diagonal line (shown as solid black line in Fig. 3). The deviation from the diagonal line towards lower side represents the over-forecasting characteristic of the prediction system. Black dashed lines refer to climatological forecast probability (vertical) and observed frequency (horizontal). Since in this study we use a percentile based (as well as actual threshold, see Methods section) definition for HW event, the climatological frequencies from forecast and observation are same. The horizontal dashed line also called as “No-resolution” line. The “No-skill” line is indicated by the red-dashed line where Brier Skill Score (BSS) become zero. The skillful region is represented by the grey coloured area^24,25^, where the BSS values are positive. Figure 3 illustrates the verification of a set of forecasts (here, it is over a 15-year period, 2003–2017), over two regions namely, NW (Fig. 3a) and SE (Fig. 3b) for different lead times i.e. wk1 (red lines), wk2 (green lines), wk3 (blue lines) and wk4 (violet lines). The sample of forecasts includes all model grid-points over the individual region selected and for the verification period. In this figure the reliability curves have positive slope (up to 70% and 80% forecast probabilities for NW and SE regions respectively), indicating that as the forecast probability of the event increases, the chance of occurrence of the event also increases. This means, the forecasts have some reliability. For the NW region (Fig. 3a), the reliability diagrams for all week leads lie within the shaded region and with positive slope up to 70% forecast probability. But, as the lead time increases the deviation from diagonal line increases, which means that, with lead time the over-prediction tendency also increases. However, it is noticed that the forecasting system is skillful in predicting the HW events (defined based on the proposed criteria) up to wk4 lead over this region, but restricted up to 70% forecast probability. But for extreme forecast probabilities, the ERP system shows no skill except wk1 lead. On the other hand, for the SE region (Fig. 3b), the reliability diagrams are skillful for all four week leads with the 50% forecast probability (though they deviate from the diagonal line as the forecast probability increases), and afterwards they cross the “No-skill” line marginally up to 80% forecast probability, illustrating it’s usefulness in decision-making. The deviation from the diagonal line (perfect reliability) towards the lower side indicates that the forecast system has a tendency of over forecasting over this region too. It can be concluded that the forecasts of HWs over NW region show good reliability compared to the SE region. So, the above skill analysis gives us the confidence to use this IITM-IMD ERP system for real-time prediction of such extreme temperature events with sufficient lead time.

### HW events over different HW prone regions

Based on the area-averaged Tmax over NW and SE regions, the HW events are identified. We have considered another region namely NorthWest-SouthEast (NWSE), to identify those HW spells having overlapping durations (at least one day) over NW and SE regions. The detailed methodology is discussed in the Methods section with an example. The HW spells over the three regions are listed in Table 1. During the period 1981–2018, twenty two events are identified over NW region, fourteen over SE region, and nine over NWSE region.

**Table 1:** List of HW events over different heat wave prone regions during 1981 to 2018.

North-West (NW)	South-East (SE)	Northwest-Southeast (NWSE)
1981: 14–21 Jun (8)	1983: 29May–03Jun (6)	1984: 17May–01Jun (16)
1985: 15–22 May (8)	1994: 07–13 May (7)	1998: 15May–06Jun (23)
1987: 03–08 Jun (6)	1997: 20–25 May (6)	2003: 26May–09Jun (15)
1988: 06–15 May (10)	1997: 28May–02Jun (6)	2005: 18May–09Jun (23)
1988: 22May–05Jun (15)	2002: 09–15 May (7)	2012: 24May–09Jun (17)
1989: 11–24 May (14)	2002: 19–24 May (6)	2013: 15–26 May (12)
1991: 31May–06Jun (7)	2008: 15–22 May (8)	2015: 18–31 May (14)
1992: 14–19 Jun (6)	2012: 16–21 May (6)	2016: 11–28 May (18)
1993: 02–08 May (7)	2014: 17–24 May (8)	2018: 17May–05Jun (20)
1993: 23–28 May (6)	2016: 10Apr–04May (25)	
1993: 05–13 Jun (9)	2017: 16–30 Apr (15)	
1994: 18May–09Jun (23)	2017: 13–26 May (14)	
1995: 27May–10Jun (15)	2018: 17Apr–01May (15)	
1996: 28May–04Jun (8)	2018: 05–13 May (9)	
1999: 26Apr–05May (10)		
2002: 03–08 May (6)		
2006: 04–09 May (6)		
2009: 16–23 May (8)		
2010: 14–19 Apr (6)		
2010: 11–28 May (18)		
2014: 02–11 Jun (10)		
2016: 03–08 Jun (6)		

The duration of each spell is given in bracket.

### Composites of Tmax (actual and anomaly from observation)

**Figure 4:**
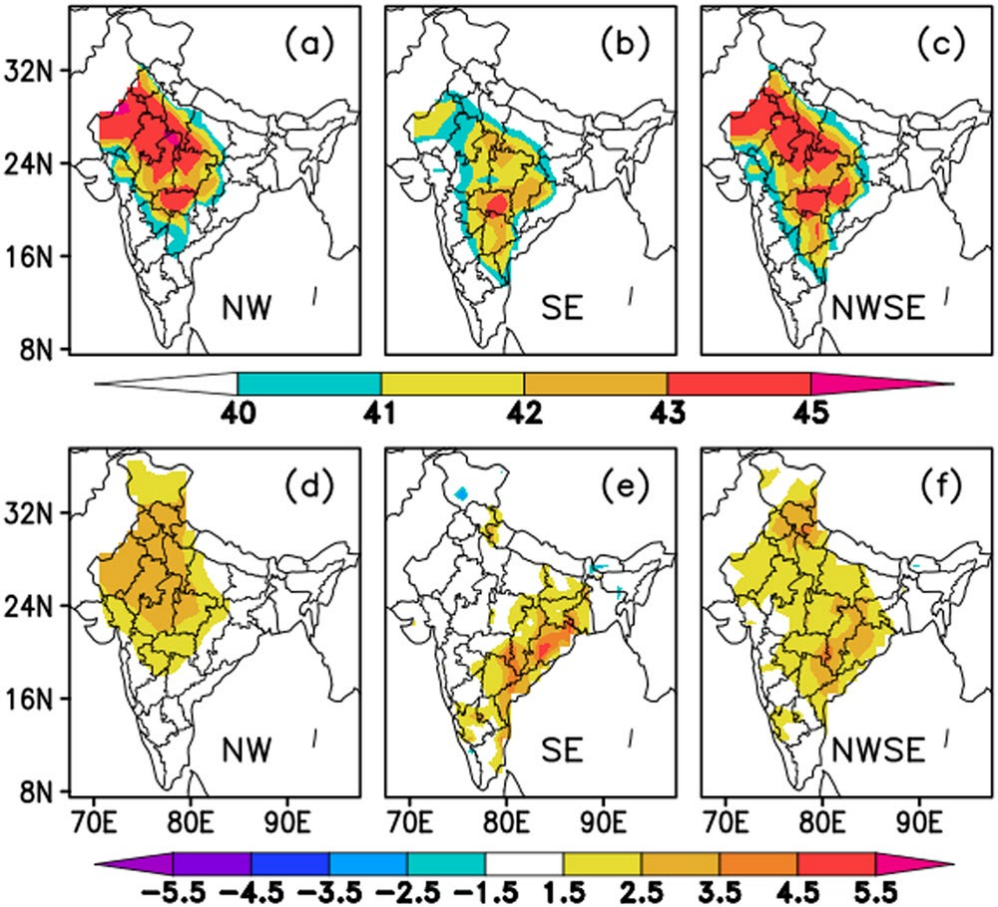
(**a**–**c**) composite of actual Tmax for NW, SE and NWSE regions respectively. (**d**–**f**) composite of Tmax anomalies for NW, SE and NWSE regions respectively.

The spatial distribution of observed actual Tmax composited for all spells in each HW prone region, NW, SE and NWSE, are shown in Fig. 4a–c respectively. It is clear from the figure that the Tmax values more than 43 °C are observed over northwest and central parts of India during HW spells over NW region, whereas during the SE-HW spells, higher Tmax values are observed over SE parts of India. In case of NWSE region, the maximum of actual Tmax is observed over northwest, central and south east regions. This spatial distribution of higher temperatures is clearer in Fig. 4d–f that depicts the composite of Tmax anomalies over those regions. Positive anomalies (departure from normal) of Tmax show high values over NW (between 2.5 – 3.5 °C), SE (more than 3.5 °C) and NWSE (ranges from 1.5 – 4.5 °C) regions for HW spells identified over those regions respectively. Based on the actual and anomalous Tmax values over the three HW prone regions, it may be suggested that, the departure of Tmax from normal plays seminal role in maintaining HWs over SE region, whereas the actual Tmax values are important in maintaining the HWs over NW region.

### Verification of the ERP for HW events

It is evident from Figs 2 and 3 that the ERP system has promising skill in predicting the extreme temperatures. The efficiency of ERP system in predicting the HW spells over Indian region is examined here through the verification of selected HW events over three HW prone regions in terms of probabilities of occurrence of HW event for different phases of the event, such as, onset, duration and demise. For the verification of onset, duration and demise phase of HWs, the first three consecutive days, total length of the spell and last three consecutive days during the spell are considered, respectively. For each phase of a HW event, the model forecasts are considered from the nearest three initial conditions (ICs). The ICs are written in MMDD format on each figure. Since the forecast are available maximum up to 32 days lead time from each IC, the duration of the longest HW spells are reduced by one or two days wherever it is required. However, the duration for both the observation and model are kept same. To choose the HW events for the verification over different regions, the observed probabilities and the intensities (defined as the averaged Tmax anomaly during the spell) of all the individual spells during 2003 to 2018 are calculated (as the hindcast is available from 2003 onwards) (please refer the Supplementary Figs S2 and S3). From these figures, those events with the high probabilities and with the high intensities are chosen, along with few cases with forecast misses. Out of them, verification of three spells (one for each HW prone region) are chosen as main figures and few are shown in the Supplementary Figures.

**Figure 5:**
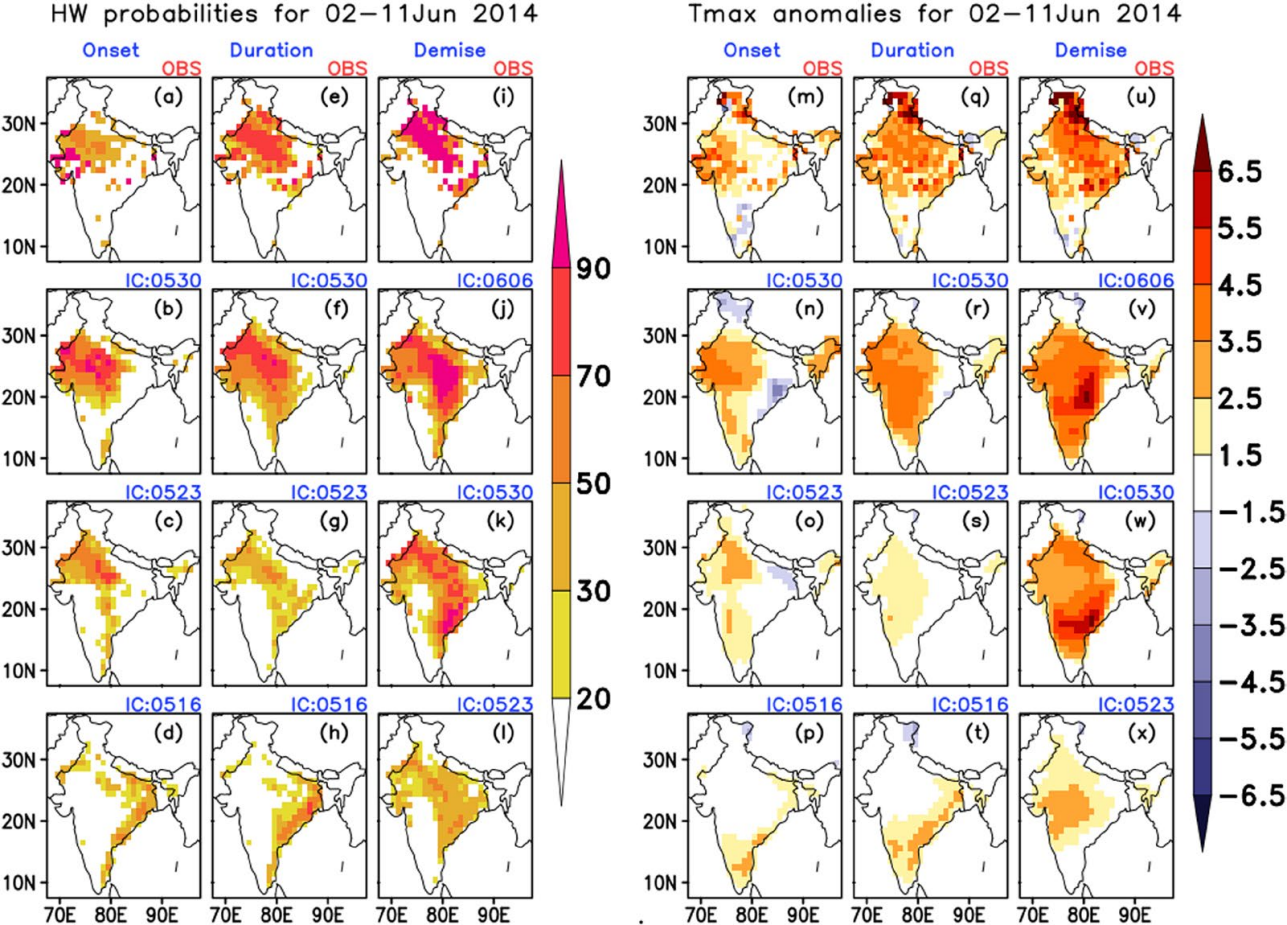
Probabilities of occurrence of HW (**a**–**l**) and average Tmax anomalies (**m**–**x**) during the HW period 02–11 June 2014. Top most panels represent the observed (mentioned as OBS on the top of the panels) and the subsequent panels represent the model predicted values for three nearest initial conditions (written on the top of each panel in the format MMDD).

Over NW region (spell: 02–11 June 2014): The year 2014 witnessed several HW spells, affecting different regions in different months and raising the death toll more than 1500 (source historical newspaper). In early June, north and north western parts of the country was hit by an intense HW spell. Late onset and weak northward progression of monsoon over India in early June^26^ might have led to clear skies conditions, which in turn resulted in persistence of high temperature, resulting in an intense HW condition. Figure 5 shows the probabilities of occurrence of HW (Fig. 5a–l) and average Tmax anomalies (Fig. 5m–x) during the heat wave period. The top most panels are for observation and subsequent panels are for model forecasts from three nearest ICs (ICs are mentioned on the top of each panel). This spell had its onset over the western parts of the country and then remained for almost 11 days over the NW region. The onset (Fig. 5a–d,m–p) was predicted very well from 30^th^ May and 23^rd^ May ICs with slightly higher probabilities from the nearest one. In case of duration (Fig. 5e–h,q–t) the ERP system was able to capture the event from the nearest two ICs reasonably well, but from 23^rd^ ICs it failed to capture the intensity. Very interestingly all the three ICs were able to predict the demise phase of the spell but with the decreasing probability and intensity (Fig. 5i–l,u–x) in farthest IC. It is also noted that, the farthest IC, 16 May, failed to predict the onset and duration of the event. This may be attributed to the reduced skill (predicting the extreme event) of the forecast system beyond wk2 lead over the NW region (as seen from Figs 2 and 3).

**Figure 6:**
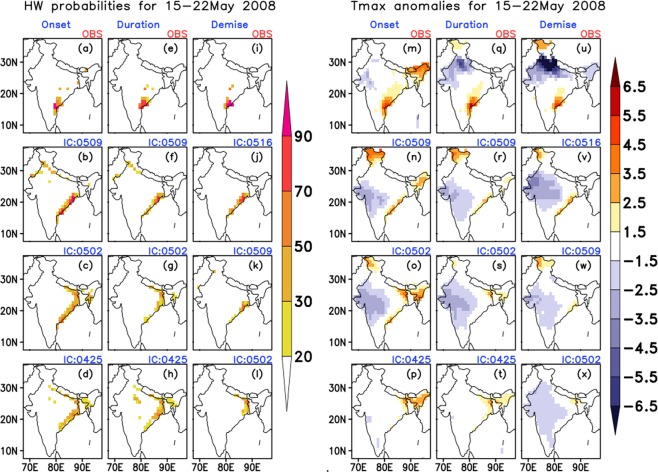
Probabilities of occurrence of HW (**a**–**l**) and average Tmax anomalies (**m**–**x**) during the HW period 15–22 May 2008. Top most panels represent the observed (mentioned as OBS on the top of the panels) and the subsequent panels represent the model predicted values for three nearest initial conditions (written on the top of each panel in the format MMDD).

Over SE region (15–22 May 2008): The coastal Andhra Pradesh and some parts of Telangana experienced a HW spell during the 3^rd^ week of May 2008. This spell is chosen as the onset, duration and demise phases of the spell are over a very localized region, with sufficient intensity. From Fig. 6a–d,m–p, it is noticed that there was a clear indication of onset of the spell over the coastal Andhra Pradesh region (with some spatial error) from 2^nd^ May IC. The ERP system could capture the duration (Fig. 6e–h,q–t) and demise (Fig. 6i–l,u–x) from 9^th^ May IC reasonably. But from farthest IC, the forecast system failed to predict the exact location of the event.

**Figure 7:**
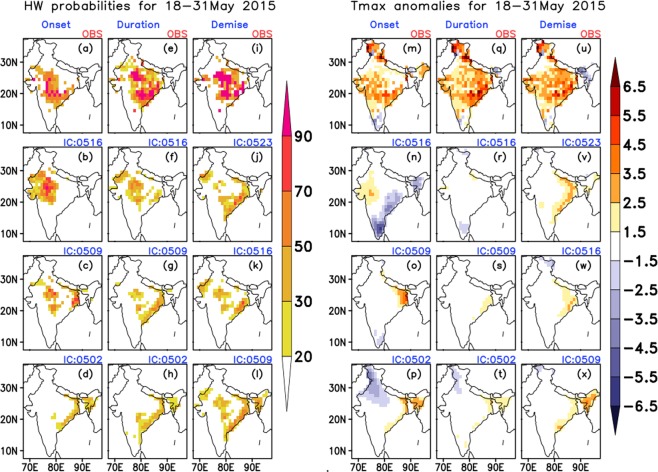
Probabilities of occurrence of HW (**a**–**l**) and average Tmax anomalies (**m**–**x**) during the HW period 18–31 May 2015. Top most panels represent the observed (mentioned as OBS on the top of the panels) and the subsequent panels represent the model predicted values for three nearest initial conditions (written on the top of each panel in the format MMDD).

Over NWSE region (18–31 May 2015): This was one of the deadliest HW events recorded in the recent years, which affected most of the parts of the country and has caused the deaths of at least 2,500 people^5,6,15^. This spell lasted for almost 14 days during the second half of May 2015. The severely affected states were Andhra Pradesh, Telangana and parts of West Bengal and Orissa (please refer Supplementary Fig. S1 for the regions). It also covered the central and northwestern parts of the country. From Fig. 7a–d,7m–p, it is noticed that there was a clear indication on the onset of this event over northwest and central parts of the country from 9^th^ May IC (with the decreasing probability and intensity with increasing lead time). The forecast system was able to capture the duration (Fig. 7e–h,q–t) as well as the demise phase (Fig. 7i–l,u–x) reasonably from all the three ICs (especially over SE coastal regions, but with less magnitude). Thus, it is noted the IITM-IMD ERP system could predict this spell reasonably well, with sufficient lead time.

## Discussion

The main goal of the ERP group of IITM is to reach to the end users by developing various customised forecast products and warning systems for the societal benefits. Once developed and verified for hindcast data, all the products will be transferred to India Meteorological Department (IMD), which is the authorized Govt. agency to issue the forecasts/warnings, to make them operational in real time. This study makes an attempt to explore the strength of the CFSv2 based MME forecast system (developed at IITM) in using a new criterion (based on gridded Tmax with 1° × 1° resolution) for the real-time ERP of HWs over Indian region during MAMJ. Since the CFSv2 model has a temperature bias over Indian land, the climatological temperature bias has been removed for Tmax before applying it in the HW criterion. This study proposes a criterion for HW using the thresholds of actual, departure from normal and the percentile values of Tmax (discussed in Methods section). Two HW prone regions are identified namely NW and SE using newly defined HW criterion. The southeastern coastal regions which are vulnerable to HWs in the recent years, are also considered in SE.

The efficiency of the ERP system in predicting the extreme temperatures is assessed by calculating the ACC, SEDI and reliability. The ACC values of Tmax up to wk2 lead show remarkable skill over most parts of the country, especially over the HW prone areas. The SEDI values are calculated for 70, 50 and 30 percentage probabilities of observing HWs. It is noticed that the ERP system is skillful up to wk2 lead in predicting the HW condition, even with 70% probability. It is worthwhile to note that the SEDI values are skillful with 50% probability of HW over few parts of NW and SE India in wk3 lead and for 30% probability, values are significant only over east and south-eastern parts up to wk4 lead. To check the reliability of the forecast system in predicting the HW events using the proposed criterion, the reliability diagrams are plotted for NW and SE regions for four different time leads. It is found that the reliability diagrams show significant skill over NW regions (up to 70% forecast probabilities) compared to the SE regions up to wk2 lead. It is found that the ERP system has tendency of over-forecasting the events over both the regions, although it is less over NW compared to SE region. Also it shows useful skill for wk3 and wk4 leads with moderate forecast probabilities. Therefore, the information contained in the reliability diagrams may be useful to make approximate corrections to the forecast probabilities. Overall, the IITM-IMD ERP system is found to be beneficial for HW prediction over Indian region with sufficient lead time.

The HW events are identified based on the area averaged Tmax values over these two regions and the thresholds are chosen according to the area averaged 95^th^ percentile values of Tmax for the individual regions. Another region, NWSE, is also defined for those HW events that exist over NW and SE regions and with the overlapping (at least one day) spell duration. From the composite analysis, it is found that the HW spells over NW region are associated with the high values of actual and anomalous Tmax over NW and central parts of India, while for SE region the Tmax (actual and anomalies) values are concentrated over south east coastal parts of the country. For NWSE region, the maximum values of Tmax are observed over most of the HW prone regions of the country except north-eastern and south-western parts.

The strength of the ERP in using the proposed criterion for predicting the HW events over India, has been verified for some important HW events over three regions (also please refer the Supplementary Figs S4–S9). It is noticed that the ERP system could reasonably predict the onset, duration and demise phases of most of the events with sufficient lead time. However, the probability decreases with increasing lead time, compared to the observed probability (also see Figs S4, S5, S8). It is also observed that there are few cases where the ERP system failed to predict the onset (over space and time) even from the nearest IC, but could capture the duration and demise phase (see Figs S6, S7). This could be linked to the spatial and temporal errors present in the ERP system. Also there are incidents of event-to-event variability such as missing some events, even from the nearest ICs (see Fig. S9), whereas at times there were indication from farthest ICs on the duration and demise of some events. It may be attributed to the uncertainty present in the model ICs and inherent spatial and temporal errors in the model in predicting the extreme events^27,28^. However, it is found that the proposed criterion has shown potential in providing an outlook on the forthcoming HW spells with sufficient lead.

As this study deals with the real-time monitoring and prediction of HW events over India by proposing a criterion for gridded data, the identification of the physical mechanisms behind these HW events are beyond the scope of the present study. It is known that relative humidity plays an important role in modulating the severity of HW spells^10,29^. Although limitations exist in the availability and quality of relative humidity observations, there is a scope for incorporating the relative humidity factor while defining HW events and their prediction. Future work will focus on the improvement of the forecast signal (specially over SE region) using some statistical post-processing techniques and on the reduction of model biases for predicting HW events with more accuracy.

## Methods

### Data

For the proposed heat wave criteria (daily basis), daily gridded (1° × 1°) IMD observed Tmax datasets (1981–2018) have been used^30^. The daily climatology is calculated during the period 1981–2010. The climatological 95^th^ percentile of Tmax is calculated from daily values during March-June and for 1981–2010. These datasets are available only over Indian land.

The IITM-IMD ERP system is a combination of different variants of NCEP’s Climate Forecast System version 2 (CFSv2). It is a multi-model ensemble prediction system^31^. It consists of four different variants of the same model with different resolutions, such as: (i) CFSv2 at T382 (ii) CFSv2 at T126 (iii) GFSbc (the stand-alone atmospheric component - GFSv2, forced with bias corrected SST obtained from CFSv2) at T382 and (iv) GFSbc at T126, all having 4 ensemble members each (total 16 members)^18,32–36^. The model hindcast runs are available for the period 2003–2017 and forecast is available for 2018. Now, this model is being run operationally at IMD once in a week i.e. every Wednesday throughout the year. The model outputs are available up to 32 days lead time from each initial condition and hence the limitation exists here to show the verification of each HW event beyond 32 days. The model predicted daily Tmax dataset with 1° × 1° resolution is used for the identification of HW days after correcting the bias (discussed in the next section). For skill analysis, the period chosen is 2003–2017 and for MAMJ season.

### Method of bias correction of model data

Before using the model forecasted daily Tmax data, the climatological bias has been removed for each day as follows:

1 $$Tma{x}_{bc}=Tma{x}_{raw}-(Tma{x}_{clim,model}-Tma{x}_{clim,observation})$$

where, *Tmax*_*bc*_ represents the bias corrected maximum temperature for a day

*Tmax*_*raw*_ represents the raw model output for that day

*Tmax*_*clim,model*_ is the model climatology (based on 2003–2017 data) for that day

and *Tmax*_*clim,observation*_ is the observed climatology (based on 2003–2017 data) for that day

### Heat wave criteria

The main goal of this study is the real-time monitoring and extended range prediction of heat waves using a multi-model dynamical ensemble prediction system developed at Indian Institute of Tropical Meteorology, India. For this, a criterion has been proposed based on the observed daily gridded Tmax datasets, which can be used for real-time prediction as well. The latest criterion for HW used by IMD^37^ is:

Heat wave will be considered if the maximum temperature of a station reaches at least 40 °C or more for Plains, 37 °C or more for coastal stations and at least 30 °C or more for Hilly regions.

(i) Based on departure from normal: when the maximum temperature departure from normal is 4.5 °C to 6.4 °C then it will be considered as HW day.
(ii) Based on actual maximum temperature: when the actual maximum temperature is 45 °C or more, then it will be called as HW day (only for Plains).

So, keeping this in background and also with the view of Fig. 1a,b, the present study proposes an objective criterion (same for different regions) for HWs over Indian region using gridded Tmax data. The proposed criterion is formulated in such a way that it can be used on both observational and model data, to identify and predict HW days. The criterion proposed in the present study is as follows:

(i) If the Tmax of a particular day is ≥ climatological 95^th^ percentile (calculated from daily values during March-June) and > 36 °C and also its departure from normal is > 3.5 °C for that day,
(ii) OR, simply when the Tmax is > 44 °C then, it will be defined as HW day.

In this proposed criterion, the actual Tmax threshold (i.e. 36 °C) is chosen objectively based on the spatial distribution of the climatological 95th percentile (calculated from daily values during MAMJ and for 1981–2010) values of Tmax (please refer Fig. 1b), so that this can be used for the coastal regions too. Srivastava *et al*.^30^ demonstrated that the annual average RMSE values (calculated for the 1° × 1° gridded data against the station data over Indian region) for Tmax are found to be around 0.5 °C over most parts of the country except few parts of southeastern and northwestern region where it is 0.5 – 1 °C (which may be even more for summer season). Also they have defined a threshold to identify the HW events over India during the season April-June, considering the Tmax departure from normal at a grid-point as 3 °C or more, whereas the threshold defined by IMD for a station is 4.5 °C. Therefore to make the proposed criteria more stringent and objective, the departure from normal of Tmax for each grid is chosen as 3.5 °C. The detailed analysis and its validation are discussed in Results section.

### Symmetric extremal dependence index (SEDI)

This index is defined as follows:

2 $$SEDI=\frac{\mathrm{log}\,F-\,\mathrm{log}\,H-\,\mathrm{log}(1-F)+\,\mathrm{log}(1-H)}{\mathrm{log}\,F+\,\mathrm{log}\,H+\,\mathrm{log}(1-F)+\,\mathrm{log}(1-H)}$$

where, *H* = *a*/(*a* + *c*) is the hit rate and *F* = *b*/(*b* + *d*) is the false-alarm rate. Again, a, b, c and d are the total number of hits, false-alarms, misses and correct rejections respectively in a 2 × 2 contingency table. The values for a, b, c and d are calculated during the season MAMJ and for the hindcast period 2003–17 as: (i) for observation: whether the proposed criteria for HW is satisfied or not for a particular day and (ii) for model: whether the proposed criterion is satisfied or not for that particular day but with a fixed probability (in percentage) of ensemble forecast. The value of SEDI ranges from −1 to +1, and positive values indicate better skill of the forecast system.

### Identification HW spells

To identify the HW spells over NW and SE regions during March-June, the area-averaged values of Tmax (from observation) over these regions are considered. To choose the thresholds, we have calculated the area averaged 95^th^ percentile values and it is found that the values are 43.7 °C and 40.3 °C respectively over NW and SE. Thus, the spells are identified when the area-averaged Tmax value exceeds 43 °C and 40 °C for a minimum of 6 consecutive days over NW and SE regions respectively. It is noticed that at times, the HW events develop over NW region and move towards SE parts or, occur simultaneously over both regions with some lag. Therefore, another region namely, NWSE is also considered in this study to separate out those HW spells present over NW and SE regions with overlapping (at least one day) durations. For example, if an event over NW region is having the duration from 01–10 May, and at same time other event is present over SE regions with duration 09–15 May or 08–15 May or 07–15 May or so, then these two spells will be considered as single spell with total duration 01–15 May over NWSE region.

## Supplementary information

Real time extended range prediction of heat waves over India
