# Supplementary material for: Real time extended range prediction of heat waves over India
Source: Sci Rep. 2019 Jun 21;9:9008. doi: 10.1038/s41598-019-45430-6 (PMC6588722; doi:10.1038/s41598-019-45430-6)
Supplement: Supplementary file 1 — Real time extended range prediction of heat waves over India [file 41598_2019_45430_MOESM1_ESM.pdf]

Real time extended range prediction of heat waves over India

Raju Mandal[1,2], Susmitha Joseph[1], A. K. Sahai[1*], R. Phani[1], A. Dey[1], R. Chattopadhyay[1] and D. R. Pattanaik[3]

[1] Indian Institute of Tropical Meteorology, Pune, India

[2] Department of Atmospheric and Space Sciences, Savitribai Phule Pune University, India

[3] India Meteorological Department, New Delhi, India

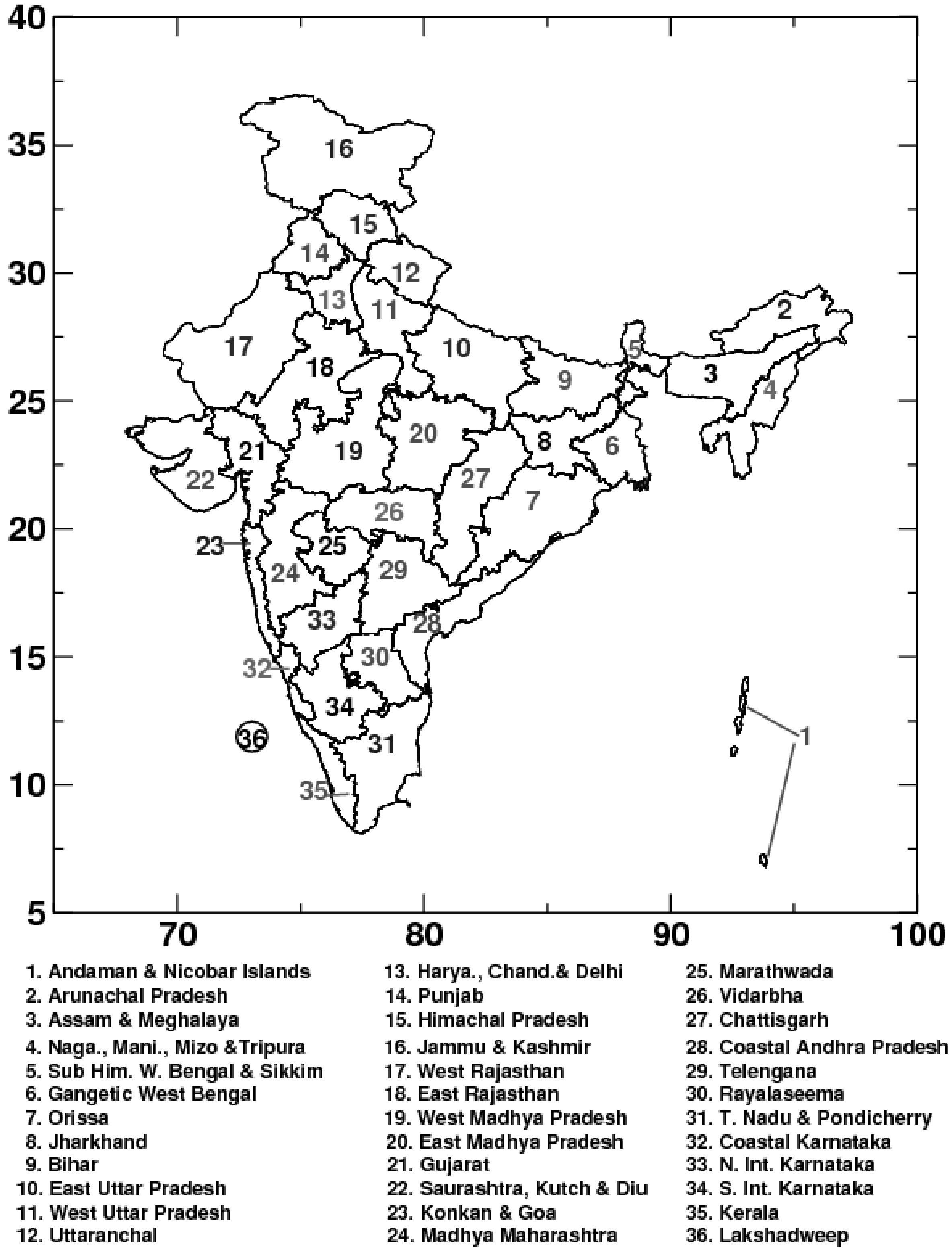

Figure S1: Different meteorological subdivisions of India.

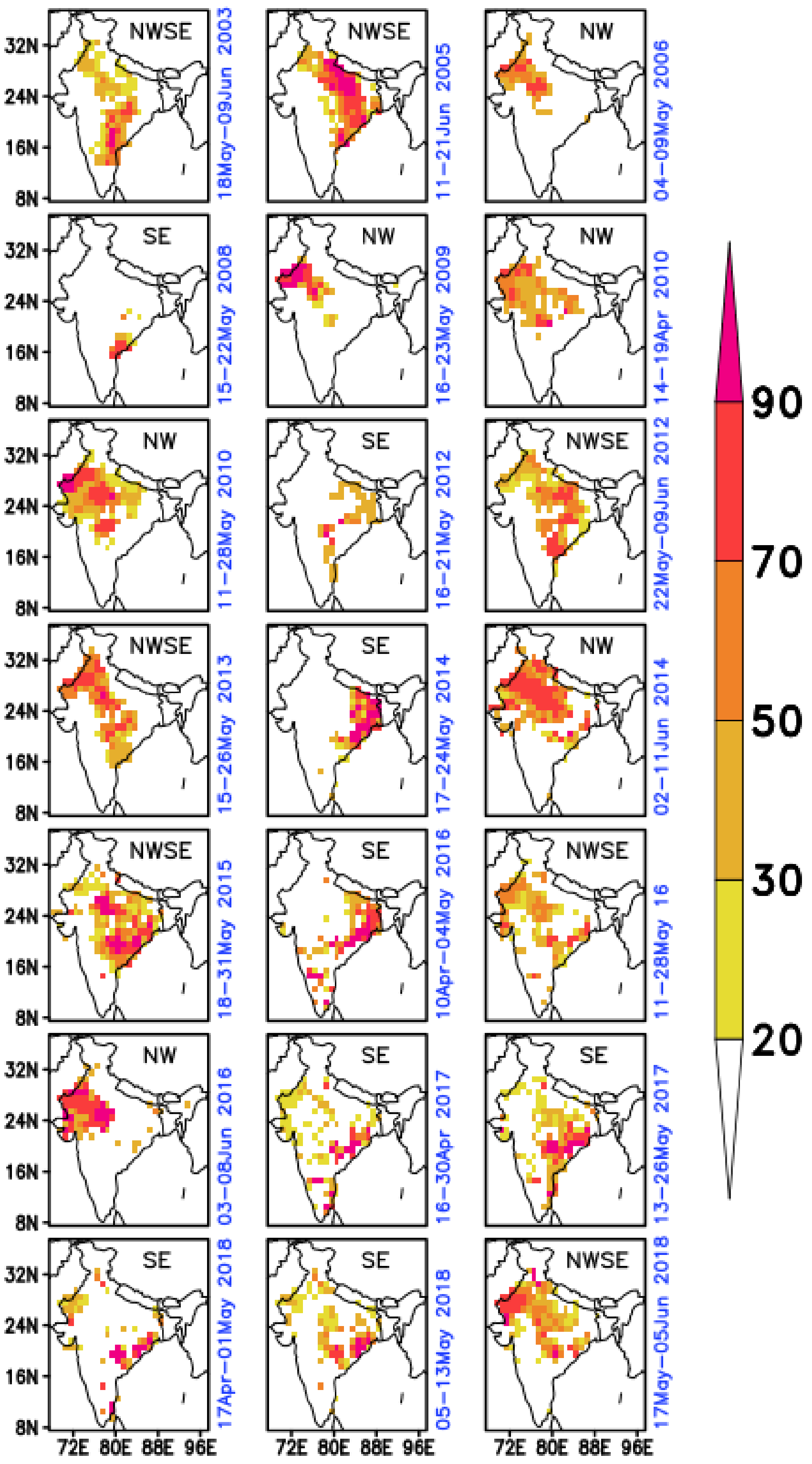

Figure S2: Observed HW probabilities during different HW spells for the period 2003-2018 over three different regions namely NW, SE and NWSE (mentioned on each panels).

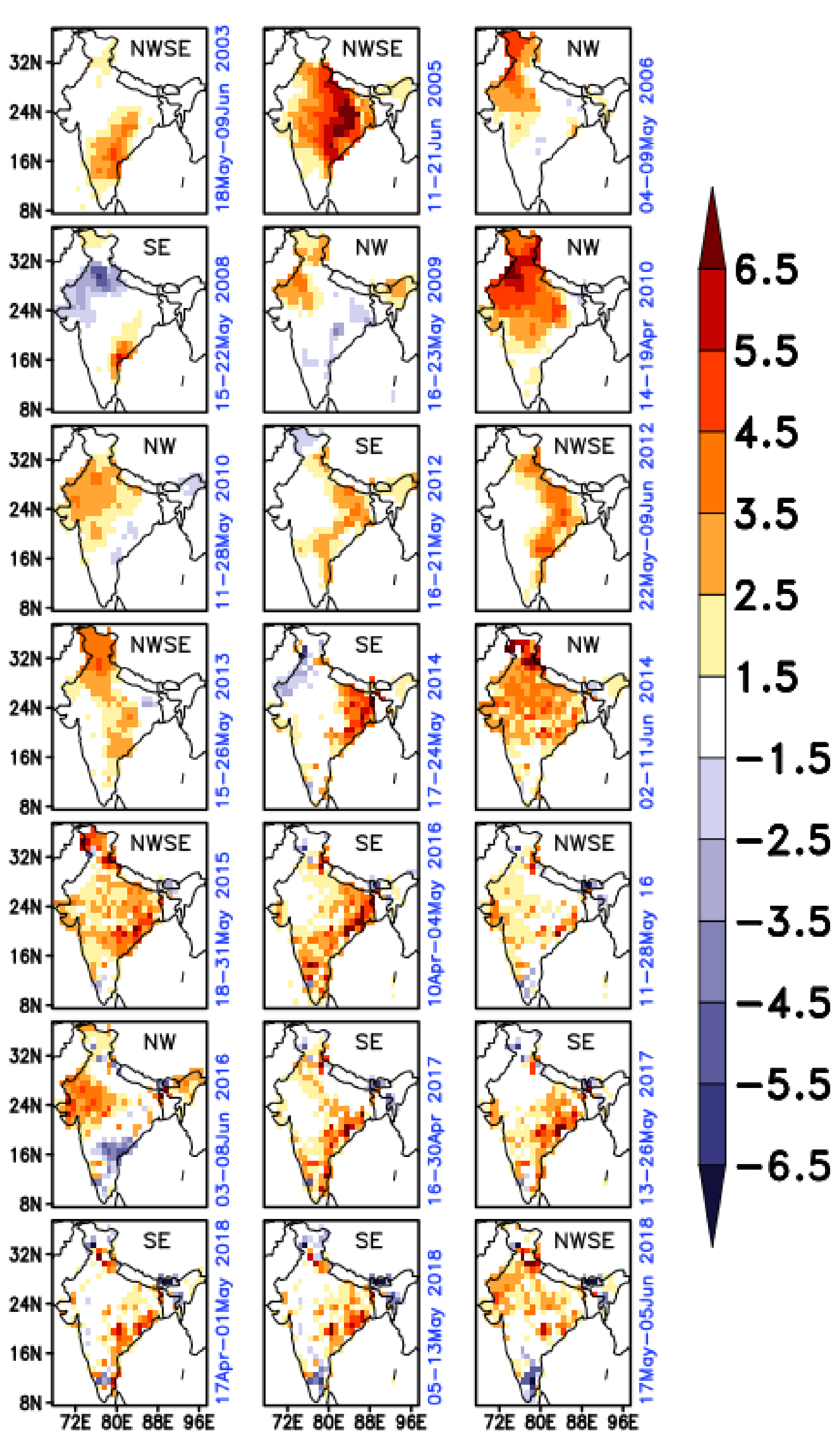

Figure S3: Observed averaged Tmax anomalies during different HW spells for the period 2003-2018 over three different regions namely NW, SE and NWSE (mentioned on each panels).

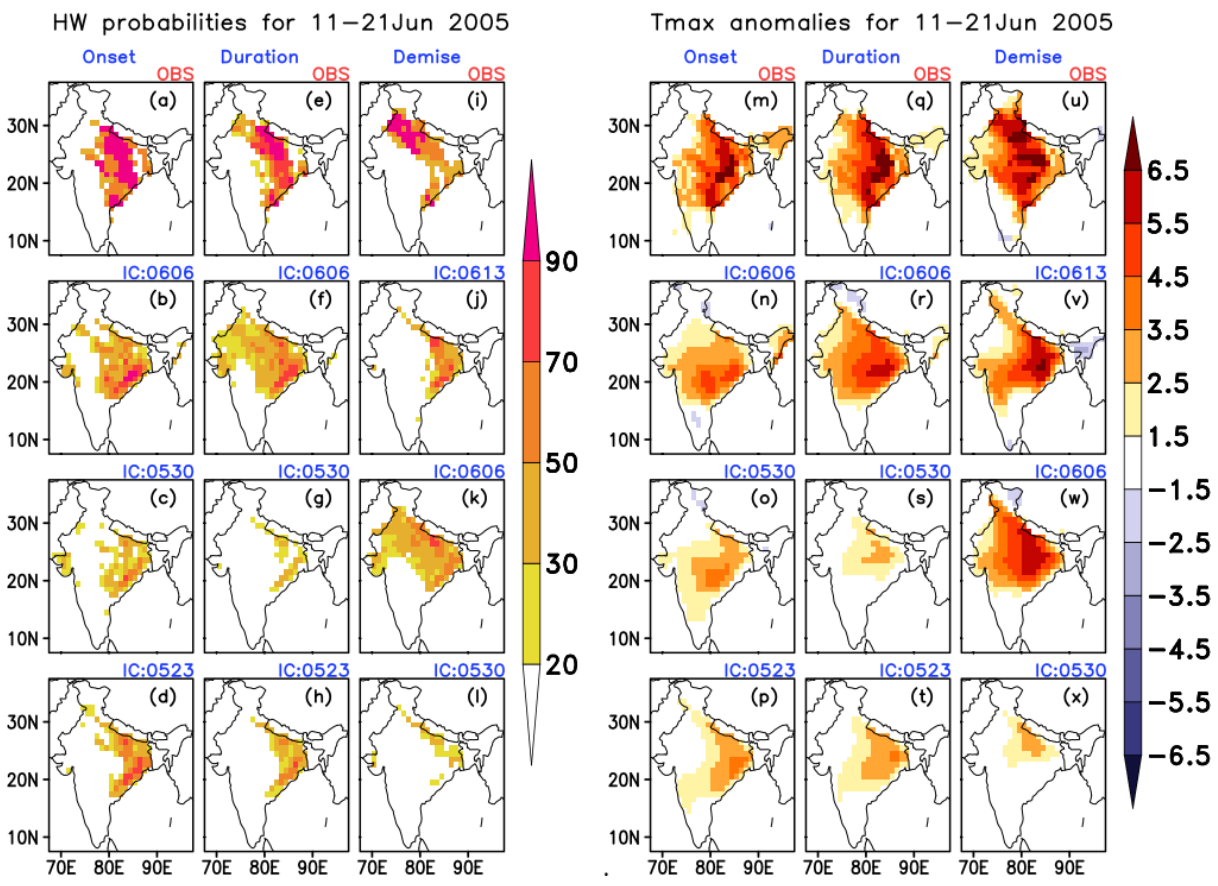

Figure S4: Probabilities of occurrence of HW (a-l) and average Tmax anomalies (m-x) during the HW period 11-21 June 2005. Top most panels represent the observed (mentioned as OBS on the top of the panels) and the subsequent panels represent the model predicted values for three nearest initial conditions (written on the top of each panel in the format MMDD).

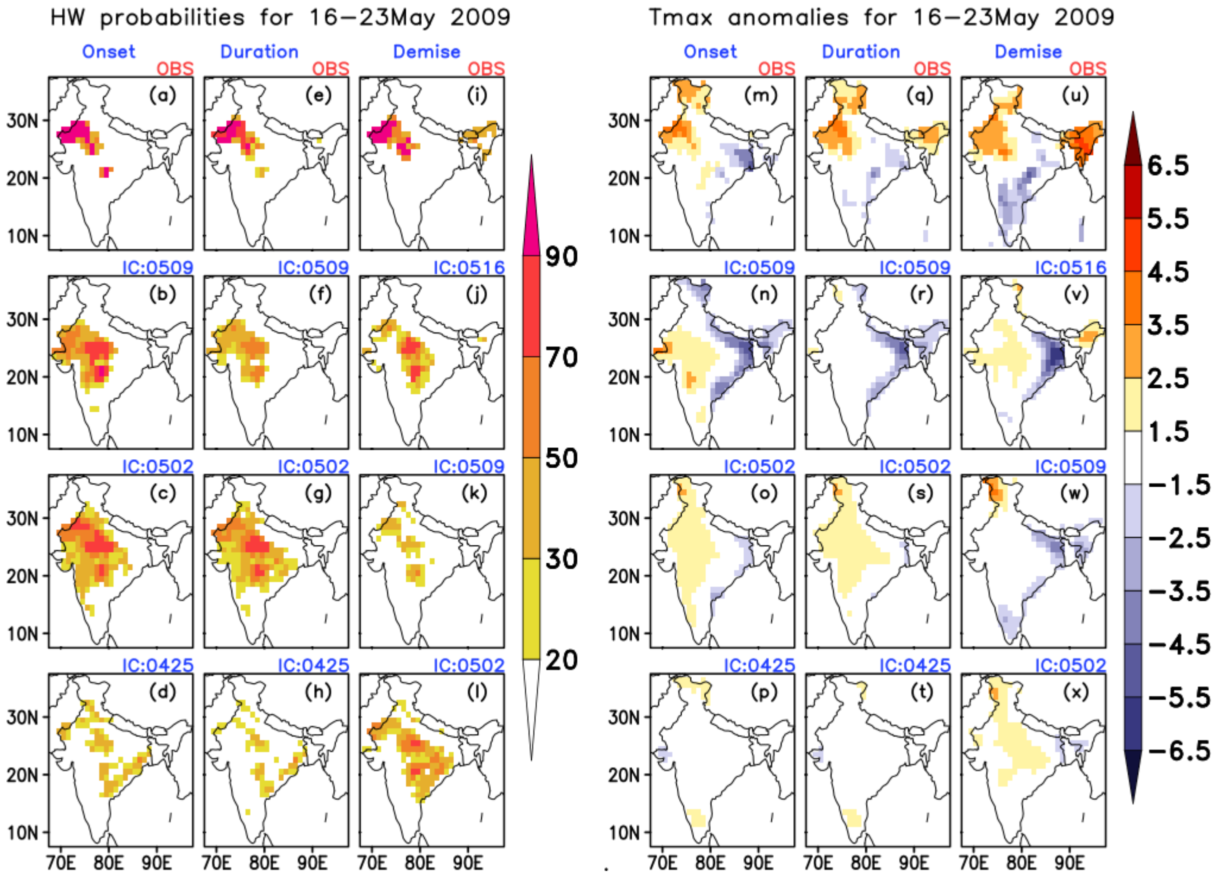

Figure S5: Probabilities of occurrence of HW (a-l) and average Tmax anomalies (m-x) during the HW period 16-23 May 2009. Top most panels represent the observed (mentioned as OBS on the top of the panels) and the subsequent panels represent the model predicted values for three nearest initial conditions (written on the top of each panel in the format MMDD).

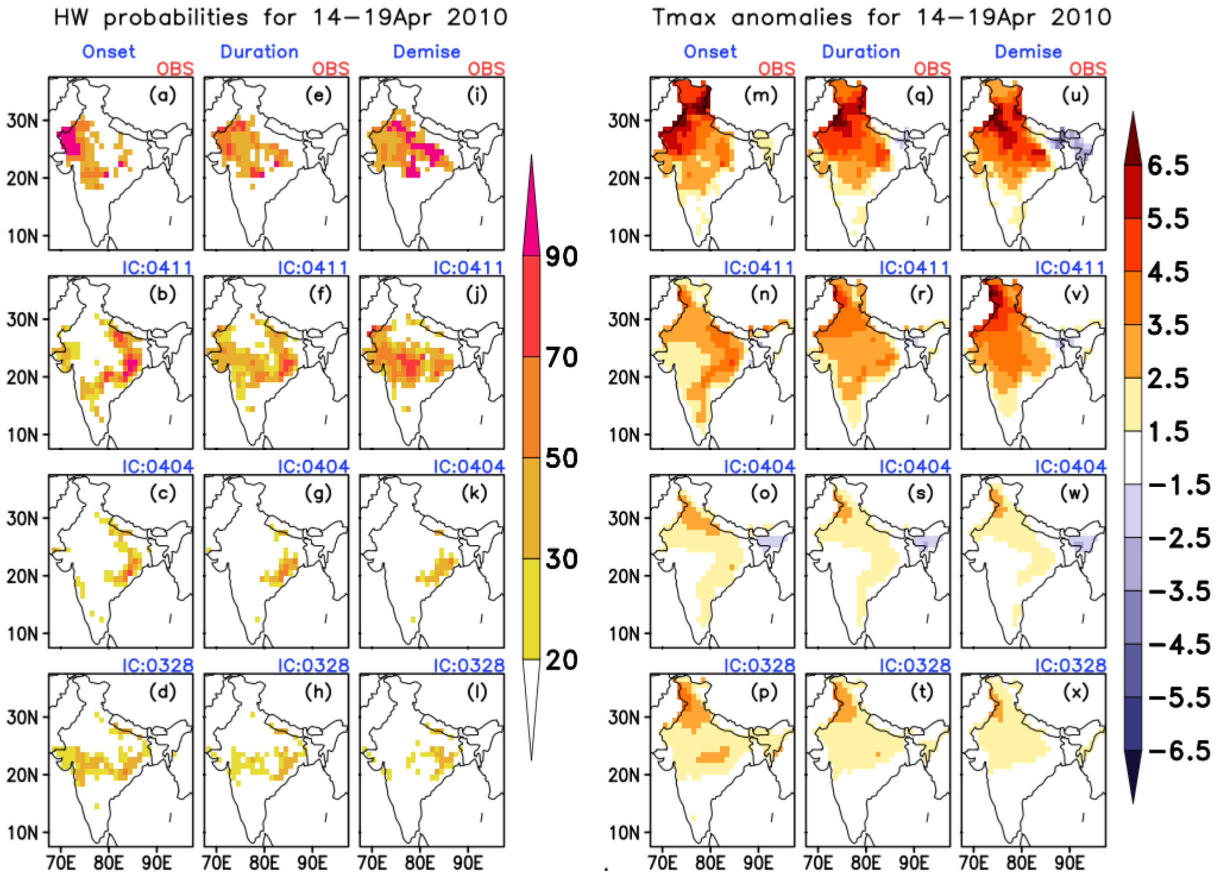

Figure S6: Probabilities of occurrence of HW (a-l) and average Tmax anomalies (m-x) during the HW period 14-19 April 2010. Top most panels represent the observed (mentioned as OBS on the top of the panels) and the subsequent panels represent the model predicted values for three nearest initial conditions (written on the top of each panel in the format MMDD).

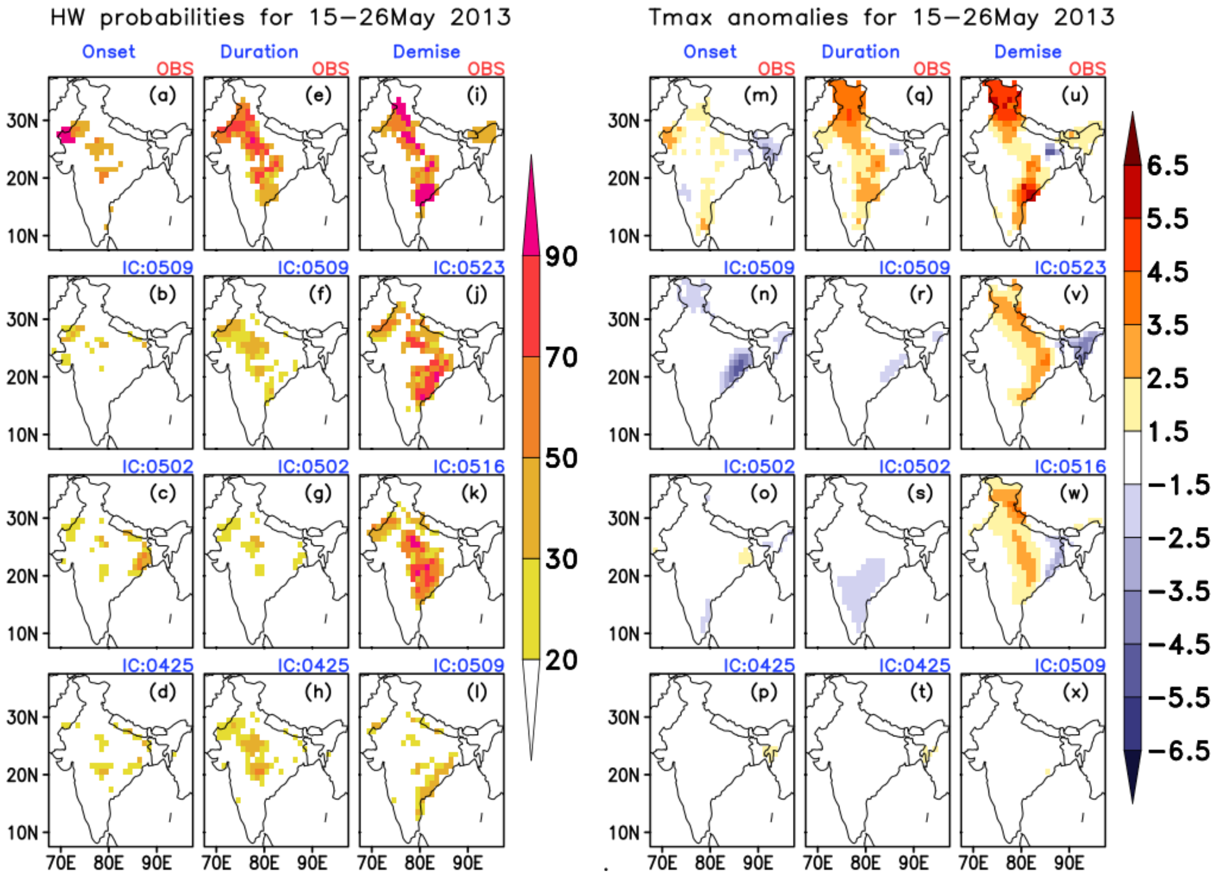

Figure S7: Probabilities of occurrence of HW (a-l) and average Tmax anomalies (m-x) during the HW period 15-26 May 2013. Top most panels represent the observed (mentioned as OBS on the top of the panels) and the subsequent panels represent the model predicted values for three nearest initial conditions (written on the top of each panel in the format MMDD).

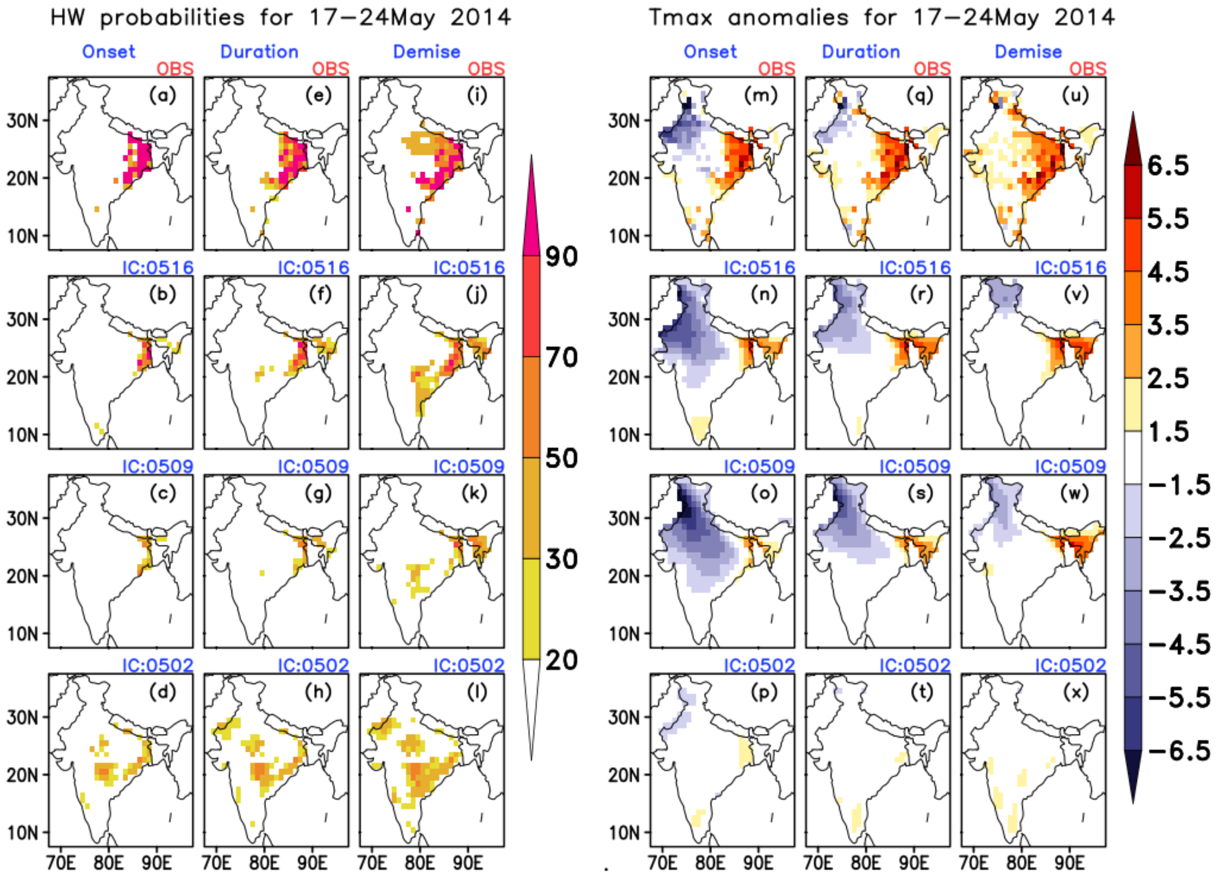

Figure S8: Probabilities of occurrence of HW (a-l) and average Tmax anomalies (m-x) during the HW period 17-24 May 2014. Top most panels represent the observed (mentioned as OBS on the top of the panels) and the subsequent panels represent the model predicted values for three nearest initial conditions (written on the top of each panel in the format MMDD).

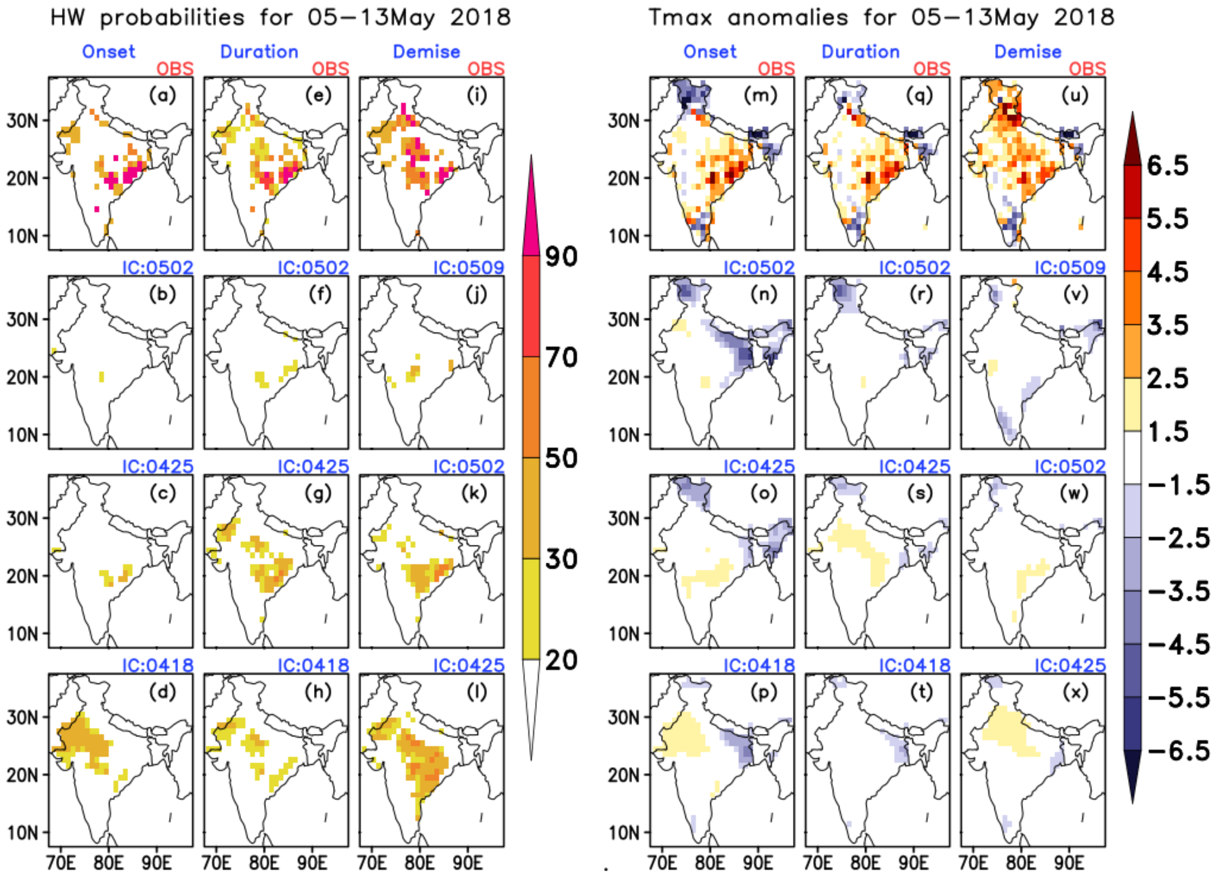

Figure S9: Probabilities of occurrence of HW (a-l) and average Tmax anomalies (m-x) during the HW period 05-13 May 2018. Top most panels represent the observed (mentioned as OBS on the top of the panels) and the subsequent panels represent the model predicted values for three nearest initial conditions (written on the top of each panel in the format MMDD).
